# Role of variant allele fraction and rare SNP filtering to improve cellular DNA repair endpoint association

**DOI:** 10.1371/journal.pone.0206632

**Published:** 2018-11-08

**Authors:** David M. Vossen, Caroline V. M. Verhagen, Reidar Grénman, Roelof J. C. Kluin, Marcel Verheij, Michiel W. M. van den Brekel, Lodewyk F. A. Wessels, Conchita Vens

**Affiliations:** 1 Division of Cell Biology, The Netherlands Cancer Institute, Amsterdam, The Netherlands; 2 Department of Head and Neck Oncology and Surgery, The Netherlands Cancer Institute, Amsterdam, The Netherlands; 3 Department of Otorhinolaryngology—Head and Neck Surgery, Hospital and University of Turku, Turku, Finland; 4 Department of Medical Biochemistry and Genetics, Turku University, Turku, Finland; 5 Genomics Core Facility, The Netherlands Cancer Institute, Amsterdam, The Netherlands; 6 Department of Radiation Oncology, The Netherlands Cancer Institute, Amsterdam, The Netherlands; 7 Institute of Phonetic Sciences, University of Amsterdam, Amsterdam, The Netherlands; 8 Department of Oral and Maxillofacial Surgery, Academic Medical Center, Amsterdam, The Netherlands; 9 Division of Molecular Carcinogenesis, The Netherlands Cancer Institute, Amsterdam, The Netherlands; 10 Department of EEMCS, Delft University of Technology, Delft, The Netherlands; University of South Alabama Mitchell Cancer Institute, UNITED STATES

## Abstract

**Background:**

Large cancer genome studies continue to reveal new players in treatment response and tumorigenesis. The discrimination of functional alterations from the abundance of passenger genetic alterations still poses challenges and determines DNA sequence variant selection procedures. Here we evaluate variant selection strategies that select homozygous variants and rare SNPs and assess its value in detecting tumor cells with DNA repair defects.

**Methods:**

To this end we employed a panel of 29 patient-derived head and neck squamous cell carcinoma (HNSCC) cell lines, of which a subset harbors DNA repair defects. Mitomycin C (MMC) sensitivity was used as functional endpoint of DNA crosslink repair deficiency. 556 genes including the Fanconi anemia (FA) and homologous recombination (HR) genes, whose products strongly determine MMC response, were capture-sequenced.

**Results:**

We show a strong association between MMC sensitivity, thus loss of DNA repair function, and the presence of homozygous and rare SNPs in the relevant FA/HR genes. Excluding such selection criteria impedes the discrimination of crosslink repair status by mutation analysis. Applied to all KEGG pathways, we find that the association with MMC sensitivity is strongest in the KEGG FA pathway, therefore also demonstrating the value of such selection strategies for exploratory analyses. Variant analyses in 56 clinical samples demonstrate that homozygous variants occur more frequently in tumor suppressor genes than oncogenes further supporting the role of a homozygosity criterion to improve gene function association or tumor suppressor gene identification studies.

**Conclusion:**

Together our data show that the detection of relevant genes or of repair pathway defected tumor cells can be improved by the consideration of allele zygosity and SNP allele frequencies.

## Introduction

Recent large-scale sequencing efforts stimulated oncology research and revealed a multitude of novel genomic alterations and somatic mutations in various tumor types [1–3]. These genetic studies are driven by the need to understand the processes related to tumor development and treatment response with the ultimate goal to find therapeutic biomarkers and targets for novel therapeutic approaches [4,5]. A major challenge in the clinical translation of these results is the discrimination of genetic alterations with a functional impact from the vast number of detected alterations.

While high gene mutation frequencies in tumors can point to potential oncogenes, defining a role for tumor suppressor genes (TSG) can be challenging. This is because TSG variants with a functional impact likely affect pathway performance only when the wild-type allele is lost. Variants in DNA repair genes, archetypal TSGs, are particularly difficult to evaluate. Many genes are involved in the repair of DNA damage and determine cellular survival following DNA damage. Mutations in any of these genes could influence cellular outcome following DNA damage. The multitude of genes and variants therefore hampers gene mutation detection as it is difficult to identify among them the affected gene or the pathway disrupting variant. Attempts to deduct DNA repair defects from genetic data for example for treatment response analyses suffer from the ignorance of the functional impact of many of the gene variants. Experimental validation of the functional impact of the individual variants is however time-consuming and costly. Rarity may also discourage thorough characterizations of the individual variant. An important role of genetic DNA repair defects in tumors could therefore been masked by a multitude of different, rare but functionally important, variants across many genes [6]. Computational tools that help to prioritize the potential functional relevance of genetic alterations are therefore of great importance. Many different tools, comprehensively reviewed in Eilbeck et al. and compared by Mahmood et al. [7,8], have been developed. They use different tactics and are valuable tools to assess a variant’s probability to affect gene function. Algorithms that predict the effect of non-synonymous variants often consider the degree of conservation of homologous sequences and how disruptive an amino acid change is based on its physical properties [9,10]. However, these approaches may not suffice and in the presence of wild-type alleles such annotations are inapt to predict cellular pathway performance such when applied to tumor suppressor or DNA repair genes.

Here we propose a simple, complementary strategy that combines the zygosity status and allele frequency of variants in order to enrich for variants that label genes or tumor cells with a functional DNA repair defect. Germline *BRCA1/2* variants that predispose to breast cancer illustrate the potential value of such approaches. Pathogenic *BRCA1/2* variants manifest their deleterious potential after loss of the wild-type allele, a process known as loss of heterozygosity (LOH) [11]. Homozygosity can thus be a selection criterion to enrich for variants with a functional impact, in particular those causing a loss of function or that mark LOH events. Pathogenic *BRCA1/2* variants occur at very low allele frequencies and are therefore often annotated in dbSNP. Variant selection protocols that remove dbSNP annotated variants in order to identify somatic mutations, also remove rare pathogenic variants [12]. To prevent this some studies select only variants that are very likely to impair gene function, such as nonsense and frameshift mutations. Other selection criteria question whether variants have a confirmed association with familial breast cancer. Such associations do, however, not reflect cellular DNA repair performance well; an endpoint relevant to treatment response association studies, in particular to targeted drugs such as to PARP inhibitors [13–15]. This is partly due to the rarity of these pathogenic variants. Also, hypomorphic gene mutations among rare single-nucleotide polymorphisms (SNPs) can influence DNA repair and response to a degree that affects drug response but not cancer incidence rates. These hypomorphic gene mutations as well as low penetrance variants will be removed by variant selection protocols that solely retain confirmed pathogenic variants. The retention of variants with low population frequency (rare SNPs) could address these issues and improve repair outcome association.

Here we set out to test the benefit of allele zygosity and SNP allele frequencies filters. Selection of homozygous variants and rare SNPs as selection criteria have been considered previously. These studies were either restricted to retrieving known cancer-associated variants [12] or performed exploratory analyses in multidrug screens without verifying causality [16]. In an effort to validate these variant selection criteria, we apply them in a setup that provides a functional link to the phenotypic effect of the selected, i.e. variant-marked, tumor cell lines. We focus on genes in the Fanconi anemia (FA) and homologous recombination (HR) pathways that are required to repair DNA crosslinks. Sensitivity to the DNA crosslinker Mitomycin C (MMC) drug is a hallmark of FA pathway defects and resulted in the identification of multiple FA pathway genes [17–20]. DNA interstrand crosslinks (ICL) pose critical obstacles in replication and this activates the FA core complex that is composed by ten FA pathway proteins. Together they function as a ubiquitin E3 ligase to mono-ubiquitylate FANCD2-I. This activation recruits endonucleases to cleave the DNA and allow translesion synthesis at the ICL affected DNA, ultimately however requiring members of the homologous recombination repair pathway to finalize ICL repair. Together, the FA and HR pathway has an important role in resolving mitomycinC induced ICLs. We therefore performed capture sequencing and variant calling on FA/HR genes in a panel of 29 patient derived head and neck squamous cell carcinoma (HNSCC) cell lines, of which a proportion was previously shown to have FA/HR pathway defects [21]. The defects were revealed by MMC response data and confirmed by additional crosslink repair function parameters [21]. The MMC response data provide a robust functional readout for crosslink repair that allowed us to assess the value of combined allele zygosity and SNP allele frequency filters to detect cellular repair defects and/or relevant genes.

## Methods

### Cell line panel

The following HNSCC patient derived cell lines were generated at the Turku University Hospital Finland between 1990–2002: UT-SCC-1A, UT-SCC-2, UT-SCC-4, UT-SCC-7, UT-SCC-8, UT-SCC-9, UT-SCC-12A, UT-SCC-14, UT-SCC-15, UTSCC-16A, UT-SCC-20A, UT-SCC-24A, UT-SCC-24B, UT-SCC-27, UT-SCC-30, UT-SCC-32, UT-SCC-36, UT-SCC-38, UT-SCC-40, UT-SCC-42A, UT-SCC-43A, UT-SCC-45, UTSCC-54C, UT-SCC-60B, UT-SCC-76A, UT-SCC-77, UT-SCC-79A and UT-SCC-90. The conditions under which these cell lines were cultured have been described previously [22,23]. The HNSCC cell line NKI-SCC-263 was established at the Netherlands Cancer Institute. Two FA patient derived fibroblast cell lines were provided by Dr. H. Joenje (Vrije University Amsterdam). Our sequence analyses confirmed the reported FANCG 1649delC and FANCA Arg951Gln mutations in these FA patient fibroblast line (EUFA636 [24] and EUFA173 [25]). These FA pathway mutated cell lines served as positive controls and provided the reference values in the MMC response data. These cell lines were not considered in the variant selection criteria assessment studies. Notably, all cell lines were cultured and tested under low oxygen (5%) conditions since high oxygen conditions affect cellular growth and fitness of repair defected cell lines. The negative control cell line was an hTERT transformed human fibroblast cell line (GM847), provided by Roderick Beijersbergen (the Netherlands Cancer Institute). Mitomycin C (MMC; Sigma Aldrich) sensitivities have been determined as described previously [21]. In brief, cell doublings were assessed and cellular survival after treatment with different concentrations of MMC was determined by live cell counting after multiple divisions (minimum 5) in a long-term growth assay. Controls were included with lower cell densities that tested and assured linearity in these assays. Survival was determined relative to untreated cells and the MMC concentration resulting in 50% survival (MMC IC_50_ value) was calculated for each cell line from third order polynomial curve fits on the growth inhibition curves of the individual independent experiments [14,21]. Data are from three to five independent experiments per cell line with 3 to 6 replicates each. Mean MMC IC_50_ values were used in analyses. MMC concentrations were adapted in the individual cell lines to assure a good coverage of data points in particular in the IC50 to IC90 inducing MMC dose range.

### Patient samples

Tumor samples were obtained after documented informed consent. Consent forms were approved by the medical ethical committee of the Netherlands Cancer Institute. Use of the material for this genetic study was approved by the institutional ART-CFMP biobank review board. Tumor samples are from 56 patients with advanced head and neck squamous cell carcinoma (HNSCC) that were enrolled in our hospital between 2001 and 2010 and obtained from fresh frozen pretreatment biopsies. Matched blood samples were not available for these samples.

### DNA capture and sequencing

Genomic DNA was isolated using the AllPrep DNA/RNA Mini Kit (Qiagen). Paired-end fragment libraries were prepared with the TruSeq DNA library preparation kit (Illumina) and target-captured with a SureSelect custom-based bait library (Agilent) targeting 556 genes (Table A in S1 File). The capture covers 1.9 Mb with about 24000 probes. Baits covered all exons (average coverage 1.5) and UTR and TSS were additionally covered in the canonical FA/HR pathway genes. DNA was washed, amplified, barcoded, pooled and sequenced on the Illumina Hiseq 2000 using a 2x75bp paired-end protocol. Sequencing reads were aligned to the human reference genome (GRCh37.55/hg19) with the backtrack algorithm of the Burrows-Wheeler Aligner 0.5.10 [26]. Potential polymerase chain reaction duplicates were removed with Picard Tools (http://picard.sourceforge.net). The average read coverage was 247. Blood samples from four healthy volunteers were sequenced to depict potential capture errors and bias and to monitor sequencing noise.

### Variant calling

Variants, both single nucleotide variants and small insertions and deletions, were called with VarScan 2.3.9 [27] in conjunction with Samtools mpileup 0.1.19 [28]. VarScan’s VarFreq (divided by 100) provided the variant allele fraction (VAF) values used in this study and represent the fraction of reads that show the individual variants. Single nucleotide variants were called when allele coverage was at least ten, the number of variant reads at least four, and the VAF at least 0.10. The same parameters were used for calling small insertions and deletions, except the minimum number of variant reads was set to ten with no VAF restriction. Single nucleotide variants adjacent to small insertions and deletions were removed. We used Annovar (version date 11-05-2016) [29] to annotate variants with the RefSeq and 1000 Genomes European august 2015 (Phase 3) databases [30]. SNPs refer to variants with an associated minor allele frequency (MAF) in the 1000 Genomes European database. All analyses and figures were restricted to non-synonymous exonic variants and essential splice site variants throughout this paper. Variants that occurred in all four healthy volunteer blood samples were removed as they were considered potential artifacts.

### Statistical analysis

In order to assess the value of the selection criteria to detect cellular crosslink repair defects, we compared ‘pathway mutated’ with ‘non-mutated’ cell lines in various pathways and gene sets. ‘Pathway mutated’ cell lines had one or more variants (potential mutations) in any of the pathway genes, the ‘non-mutated’ had none (Figure A in S1 File). The association between pathway mutation status and MMC response was evaluated with the Wilcoxon rank-sum test and visualized by the Positive Predictive Value (PPV). The Wilcoxon rank-sum test compares the (ranked) MMC IC_50_ values of ‘pathway mutated’ and ‘non-mutated’ cell lines. The PPV was calculated as the fraction of ‘FA/HR pathway mutated’ cell lines present among the ten most MMC sensitive cell lines (Figure A in S1 File). The number of LOH events across TSGs and oncogenes was compared with the Wilcoxon rank-sum test. Fisher’s exact test was used to compare the proportion of heterozygous and homozygous variants between TSGs and oncogenes. All statistical analyses were performed in the R environment for statistical computing.

## Results

### Variant allele fraction filtering improves functional outcome association

Here we set out to investigate the association between the zygosity status or allele frequency of a variant and its ability to mark a phenotypic effect. To this end we employed a panel of 29 patient-derived HNSCC cell lines. We employed response to the DNA crosslinker MMC as a hallmark and readout of cellular DNA repair proficiency (Figure B in S1 File). Tumor cell DNA repair capacity is the essential functional endpoint in cancer treatment response or DNA repair targeting strategies studies. Cellular MMC response is strongly influenced by the activity of the FA and HR gene products [17–20]. Assay performance and MMC sensitivity to repair relation was confirmed by the revealed MMC hypersensitivity of FA patient fibroblast controls with confirmed FA gene mutations and lack thereof in wild-type fibroblasts (Figure B in S1 File). Capture sequencing data on 556 genes, which included the canonical FA/HR pathway genes (Table A in S1 File), was available and we performed variant calling with different parameters on those genes.

**Fig 1:**
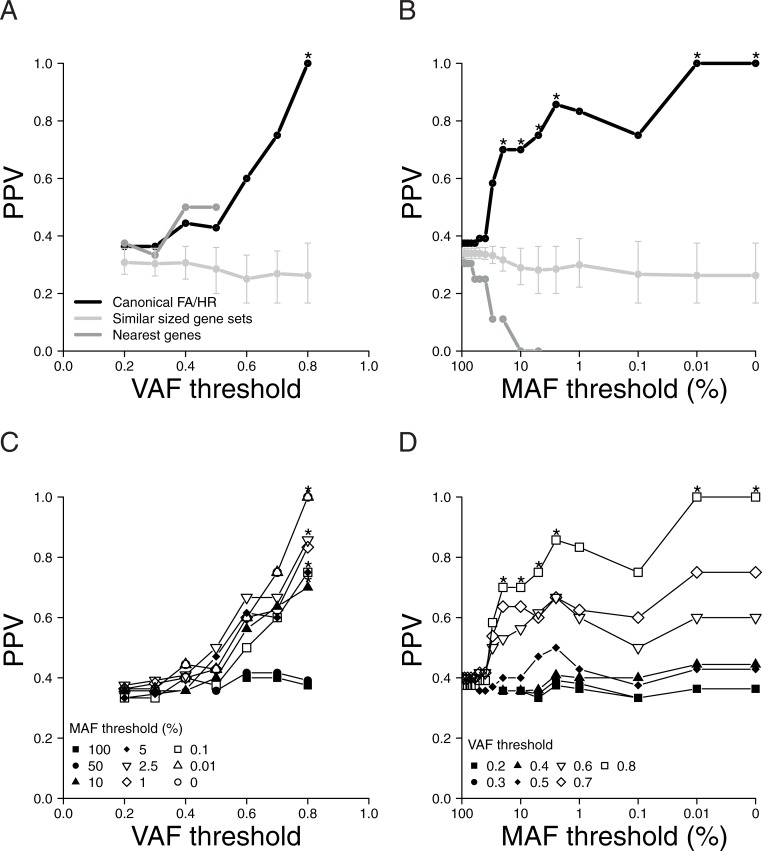
High VAF and low MAF selection criteria improve functional association. The positive predictive value (PPV) for MMC sensitivity was used to quantify the ability of variants to mark repair defected cell lines, i.e. ten most MMC sensitive, by the different variant selection criteria. Statistical analyses were omitted at sample sizes of a group of less than two and these data points and lines have been excluded in the figures. Asterisks mark a significant association with MMC response. **(A)** PPV values at each VAF threshold that was applied for variant selection are shown. Lines show the results for the canonical FA/HR genes and of the two controls: the “nearest genes” and the 10.000 similar sized randomly grouped gene sets. Error bars delineate the first and third quartile from the median in the latter. **(B)** PPV with progressively decreasing maximum MAF thresholds. Line coloring is identical to Fig 1A. **(C)** Impact of canonical FA/HR gene variant selection filters on PPV when combining multiple maximum MAF thresholds (as indicated in in-figure legend) with increasing minimum VAF thresholds (x-axis). **(D)** Influence of filters on PPVs after applying decreasing maximum MAF thresholds for multiple minimum VAF thresholds for canonical FA/HR gene variant selection as indicated.

We first factored out the allele frequency by removing all SNPs present in the 1000 Genomes database and focused on the role of the zygosity status. To this end we employed variant allele fraction (VAF) as a measure of allele zygosity, with high VAF corresponding to homozygosity. The VAF distribution of all non-synonymous variants reflected allele zygosity well in these cell lines (Figure C in S1 File). We varied VAF thresholds from low to high and at each threshold value removed variants with a VAF below the threshold. Thus, each VAF threshold resulted in a unique set of variants being retained. A cell line was assigned as ‘FA/HR pathway mutated’ if any FA/HR gene variant in that cell line was retained under the tested threshold. Increasing VAF thresholds gradually reduced the number of retained variants and number of cell lines with a ‘FA/HR pathway mutated’ assignment (Figure D in S1 File). We then tested whether the MMC IC_50_ values showed an association with the ‘FA/HR pathway mutated’ status. Specifically, we performed the Wilcoxon rank-sum test and calculated the Positive Predictive Value (PPV), i.e. the fraction of ‘FA/HR pathway mutated’ cell lines present among the ten most MMC sensitive cell lines. As control we repeated our analysis on 10.000 random gene sets, each having cumulative sequence lengths similar to the FA/HR gene set (‘Similar sized gene sets’). The association between ‘FA/HR pathway mutated’ and MMC sensitivity increased and reached significance with higher VAF thresholds (Fig 1A and Figure D in S1 File). In contrast, the PPV of the control sets remained constant at 0.3, indicating a random assignment hence a lack of discrimination power (Fig 1A and Figure E in S1 File). This indicates that the specificity of FA/HR variants to detect MMC hypersensitive, and therefore repair-defected, cell lines improves with increasing homozygosity.

Next, we assessed indirect factors that could have caused or confounded the here observed association between homozygosity of FA/HR variants and MMC sensitivity. The apparent association with homozygosity of the FA/HR variants could be an indirect consequence of an increased total or homozygous variant load in these MMC sensitive cell lines. However, neither the total nor the homozygous variant load was associated with MMC sensitivity (Figures F and G in S1 File), regardless whether SNPs were included or excluded. Homozygous FA/HR variants could act as ‘tags’ for other homozygous variants in genes in the proximity of the FA/HR genes (Table A in S1 File). However, we find that variants in neighboring genes were not associated with MMC sensitivity (Fig 1A and Figure E in S1 File, “Nearest genes”). LOH events, across a larger region neighboring and containing the FA/HR genes, are thus unlikely to have caused the association between the homozygosity of FA/HR variants and MMC sensitivity. These results show that the improved MMC response association by these filters is FA/HR gene-specific. Together, our results support homozygosity as a variant selection criterion.

### Rare SNP filtering improves functional outcome association

We next investigated the benefit of considering the allele frequency of FA/HR variants for DNA repair defect marking. We factored out the variable zygosity status by only using homozygous variants. This analysis was similar to the previous, except that variants were now filtered according to their allele frequency in the 1000 Genomes database instead of their VAF in the cell line sample. We did so by varying the allele frequency, also known as the MAF, threshold (Figure H in S1 File). The number of variants and cell lines with a ‘FA/HR pathway mutated’ assignment decreased pronouncedly at the MAF threshold value of 0.1. Notably, the association between FA/HR variant marked cell lines and MMC sensitivity increased and reached significance with lower MAF thresholds (Fig 1B, Figures H and I in S1 File). This effect was neither seen in the ‘nearest genes’ nor in the 10.000 similar sized gene sets.

We further studied the interaction between zygosity and allele frequency by filtering on both the VAF and the MAF. We then evaluated the association of the cell lines classified as repair defected by the remaining variants with MMC sensitivity. This analysis shows that the PPV increases proportionally to the VAF threshold, though only after common SNPs have been removed, i.e. at lower MAF thresholds (Fig 1C and 1D). Multiple of the VAF and MAF threshold combinations show a significant association with MMC sensitivity, further illustrating the value of a combined approach (Figures J and K in S1 File). In addition, when assessed in the two FA patient derived fibroblast cell lines, these filters identified the reported FANCG 1649delC and FANCA Arg951Gln mutations. In summary, these results show that the variant selection criteria allele zygosity and allele frequency can be combined to enrich for variants that mark cell lines with a loss of cellular repair function. Selecting homozygous variants and rare SNPs detects variants with a link to loss of pathway function in our dataset, and thus also helps to prioritize variants for further experimental validation.

### VAF and rare SNP filtering improve pathway identification

The previous analyses focused on the FA/HR pathway, because defects in FA/HR genes are well-known to impair the repair of crosslinks induced by MMC. In practice, the aim of genetic analysis is often to reveal novel associations with drug response, e.g. in pharmacogenomic interaction studies [5,31,32]. Given the importance of the FA/HR pathway for the removal of MMC induced crosslinks, the prioritization of this pathway over others by variant selection strategies that consider homozygosity and rare SNPs would confirm their value.

**Fig 2:**
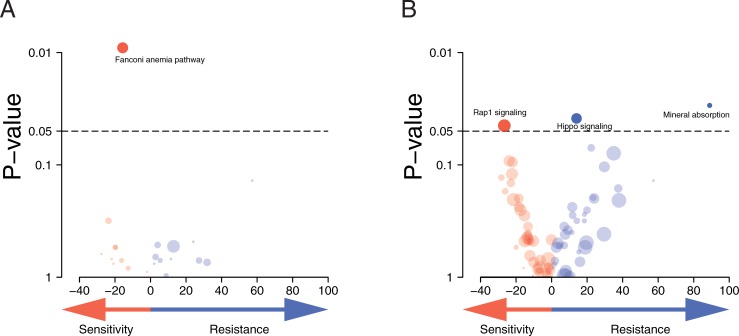
Homozygous and rare SNP variant selection criteria identify the KEGG Fanconi anemia pathway to be strongly associated with MMC response. Volcano plot showing the significance of the associations between MMC response and ‘pathway-mutation’ classification in the individual KEGG pathways as determined by the presence of homozygous and rare SNP variants in these pathway genes. The x-axis shows the difference in mean MMC IC_50_ between ‘pathway mutated’ and ‘pathway non-mutated’ cell lines. Pathway mutated cell lines are those with one or more variants in any gene of the individual KEGG pathway. The y-axis shows the significance values (Wilcoxon rank-sum test *p*-value) of the difference that were found in the MMC IC_50_ between ‘pathway-mutated’ and ‘non-mutated’ cell lines. Dot size is proportional to the number of ‘pathway-mutated’ cell lines, dot color intensity proportional to significance value. **(A)** Results after applying the variant selection strategy that selects homozygous variants (VAF ≥ 0.8) and includes rare SNPs (MAF ≤ 1%). **(B)** Results of analyses that select variants regardless of zygosity status, including rare SNPs (MAF ≤ 1%).

To this end we broadened the analysis to include all genes annotated in all Kyoto Encyclopedia of Genes and Genomes (KEGG) pathways. Based on the results of the previous section we selected homozygous variants (VAF ≥ 0.8) and included rare SNPs (MAF ≤ 1%, a threshold commonly used to define rare variants). We scored a pathway as ‘mutated’ when any of the genes annotated to that pathway were ‘mutated’, i.e. has a variant as selected by the above criteria. For each pathway, the MMC IC_50_ values of ‘pathway-mutated’ cell lines were compared to those of ‘non-mutated’ cell lines. Some mutational profiles of pathways correlated strongly (Pearson correlation ρ ≥ 0.75) because they were only represented by a few overlapping genes in our capture-set. In these cases the pathway with the weakest MMC response association was removed. The results of this analysis are represented in Fig 2A. We find that the Fanconi anemia (FA) pathway is the only pathway that is significantly associated with MMC sensitivity.

We then compared this analysis to an approach that includes all non-synonymous variants regardless of zygosity status, i.e. performs no VAF filtering. At the same time, variants and SNPs with a MAF ≤ 1% were retained. This approach was not able to reveal an association between the Fanconi anemia pathway and MMC sensitivity. This result is consistent with the requirement of loss of the wild-type, the functional DNA repair pathway gene allele, to impact cellular repair. It thereby confirms the benefit of using zygosity as a variant selection criterion to depict repair defected cells. The analysis did, however, reveal significant associations between four other pathways and MMC resistance (Figure L in S1 File). Upon further examination we found that two of these associations were mainly driven by falsely assigned *TP53* mutation status in the cell lines. Two *TP53* mutations were missed in MMC sensitive cell lines; one point mutation remained undetected because of low read depth and one known medium sized deletion was not picked up due to limitations of short-read sequencing data (Table B in S1 File) [22,33–37]. When correcting the *TP53* mutation status these two associations disappear (Fig 2B). The other two pathways associated with MMC resistance were the Hippo signaling and mineral absorption pathways, while the Rap1 signaling pathway was associated with MMC sensitivity (Fig 2B).

We next compared homozygous and rare SNP variant selection strategies to standardly used variant selection methods such as REVEL and CADD. Variants in FA/HR genes that were selected by these methods did not show an association with the functional endpoint (MMC response) at any threshold (Figure M in S1 File). These variant selection methods did also not reveal a FA pathway link in the broader pathway analysis (Figure N in S1 File). Our proposed homozygous and rare SNP variant selection strategies however did prioritize the FA pathway over all other KEGG pathways (Fig 2A), since it had the strongest association with MMC response. As the FA pathway has an established role in cellular MMC response, this supports the value of our variant selection strategy for exploratory analyses.

### Tumor suppressor genes are enriched for LOH and homozygous variants

After focusing on DNA repair genes, we next investigated TSGs in a broader sense and assessed the potential value of the homozygosity criterion for tumor suppressor gene identification in patient tumor samples. Sample tumor purity, intra-tumor heterogeneity and (aneu)ploidy hamper the ability to distinguish between heterozygosity and homozygosity based on the VAF values in tumor samples [38]. We therefore questioned whether selecting homozygous variants in tumor samples would be able to enrich for tumor suppressor genes. Homozygosity, often through the loss of a functional wild-type allele, is one of the means to accomplish the loss of the cellular function of a gene, a requirement common to TSGs. Conversely, the activating mutations in oncogenes do not necessarily require homozygosity to have a functional impact. We therefore tested whether known TSGs were enriched for LOH events/homozygous variants in patient tumor samples.

**Fig 3:**
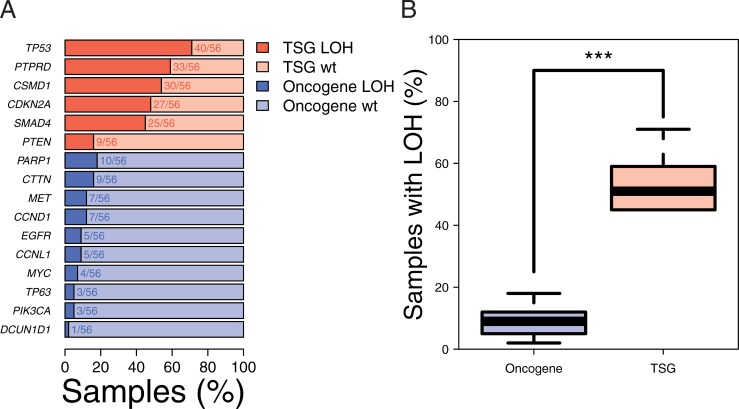
Loss of heterozygosity events are more common in tumor suppressor genes. (A) The percentage of HNSCC samples with LOH per TSG (dark red) and OG (dark blue). HNSCC TSG and oncogenes (OG) are as reported by Leemans et al. Light colors represent the percentage of samples without LOH (wt). Fraction of samples with LOH are indicated by numbers and dark colors. (B) Boxplot representation of the percentage of HNSCC tumor samples with LOH in any TSGs or OGs as shown in Fig 3A. TSGs are enriched for LOH events (*p*<0.005).

To this end, we applied the same variant calling pipeline that was used for the HNSCC cell line panel to capture sequencing data from 56 HNSCC tumor samples. Rare non-synonymous SNPs were included (MAF ≤ 1%). We find that the VAF distribution has three peaks in the tumor samples, in contrast to the bimodal VAF distribution of the HNSCC cell line panel (Figure O in S1 File). It illustrates how the contribution of tumor purity, intra-tumor heterogeneity and ploidy to the VAF values complicates the distinction between heterozygous and homozygous variants based on VAF values. We therefore used the algorithm PureCN [39] to identify genes and variants that underwent LOH. PureCN employs VAF and read coverage to produce an improved estimate of the allele specific copy number and thus LOH. Six documented HNSCC TSGs and ten oncogenes (Table C in S1 File) were used to narrow the analysis [40]. First, we determined the number of samples with LOH per gene (Fig 3A). LOH occurred five times more often in TSGs than oncogenes (average of 27 LOH events across TSGs versus 5 across oncogenes; *p*<0.005; Fig 3B). Second, we pooled the variants of all samples in the TSGs and oncogenes and calculated the fraction of gene variants that was homozygous. The fraction of homozygous variants was six times higher in the TSGs than in the oncogenes (37 / 68 versus 1 / 11; *p*<0.005).

The results show that in the HNSCC tumor samples, LOH and homozygous variants are more common in TSGs than oncogenes. This is in line with the requirement of loss of the wild-type allele for TSGs. These data indicate that selecting homozygous variants and rare SNPs could indeed enrich for genes or variants linked to a loss of function event. This variant selection strategy is therefore expected to improve associations with functional endpoints.

## Discussion

Here we tested whether variant selection strategies that consider allele zygosity and allele frequency help to mark tumor cells with functional defects. To this end we used 29 HNSCC cell lines with confirmed DNA crosslink repair defects as assessed by functional assays. We applied these strategies to the selection of variants in FA/HR pathway genes that govern crosslink repair and found a benefit of selecting homozygous variants and of retaining rare SNPs. The results show that the presence of homozygous variants and rare SNPs in the FA/HR genes is associated with functional outcome, i.e. repair defect, in the cell lines. This highlighted the potential to mark such defects in tumor cells. To demonstrate the value of our approach for exploratory analyses, we extended it to all KEGG pathways. We found that the functional outcome association is strongest in the KEGG Fanconi anemia pathway. This exemplifies the value of the variant selection criteria to identify genes or pathways relevant to a given functional endpoint. Finally, clinical application feasibility was demonstrated in the HNSCC patient tumor analysis. The enrichment of LOH events and homozygous variants in TSGs indicates improved functional association, further highlighting the potential benefit of such variant selection strategies.

Cancer genomics studies often use SNP databases to remove germline variants, but seldom use them to incorporate rare SNPs in their analysis [12]. Guiding variant selection by allele zygosity is even less customary in cancer genomics, even though this approach has successfully been used to identify pathogenic germline variants in congenital disorders [41,42]. A possible reason why this approach was underused in cancer research, is the complexity of accurately calling homozygous variants caused by LOH in tumors due to intra-tumor heterogeneity, aneuploidy and normal cell admixture [38]. Recently developed algorithms have made progress in meeting this challenge, and the identification of homozygous variants caused by LOH in tumors is now possible [38,39]. Indeed recent studies further point to the importance of considering LOH events and/or homozygosity for marking tumors with HR defects [43,44].

The selection of rare SNPs has been proposed before and was applied previously in order to capture known tumor-associated variants [12]. Since the authors acknowledged the importance of LOH, zygosity and rare SNP criteria have been also applied in attempts to identify genetic DNA repair defects [16]. However, their validity or effectiveness in depicting repair defects has formally never been tested nor confirmed. Here we therefore investigated whether such selection criteria would improve the detection of cell lines with a functional defect or the identification of the relevant genes in repair defected cell lines. In comparison to previous pharmacogenomic projects [5,31,32,45] our cell line panel data provides a unique and reliable model system for studying the effect of variant selection methods on functional outcome association. This is due to a relatively large number of uniformly treated cell lines of the same cancer type and the choice of a robust functional read out, i.e. long-term growth assays to determine MMC IC_50_ values. The commonly used short-term survival assays often reflect a mixture of growth delay and kill. They are also affected by the apoptosis proficiency of a cell line. In contrast, long-term assays reflect the overall repair proficiency, and therefore survival, better. Our chosen drug-doses also ensured that MMC IC_50_ values were reached experimentally rather than estimated by extrapolation. This is a possible improvement to the approach of others [31,32], whose IC_50_ values are discordant [46]. Unfortunately, it was not possible to extend our analysis to the publically available cell line data due to various reasons: lack of publicly available HNSCC cell line data [45], robust MMC response data [32], or DNA sequence information of the canonical FA/HR pathway genes [31]. Two resistant cell lines showed ATP7A variants, while the Hippo pathway depiction was mostly driven by APC and CTNNB1 gene variants and this points to a wnt signaling pathway contribution. A link to platinum drugs has been reported for ATP7A [47]. The validation of the possible role of the wnt signaling pathway in determining MMC resistance is however inhibited by the lack of long-term MMC survival assay data in independent HNSCC cell line panels.

While our analyses showed how the selection of non-synonymous variants, homozygous and rare as SNPs, can identify the genes and pathways responsible for the functional endpoint in question (here the FA/HR pathway for MMC response), it does not show that the selected variants cause the repair defect. This may be the case for a fraction of the selected variants. Others, however, may have simply marked LOH events in the respective genes. As rare SNPs are unlikely to be present in a homozygous state, selection of homozygosity will therefore mark LOH events and point to gene alterations that are more directly related to the repair defect / functional endpoint. Yet, with respect to the identification of variants with a functional impact, a similar selection procedure did enrich for variants with an annotation in COSMIC or predicted to alter protein function by other algorithms in our previous study [21]. After accounting for the individual stroma component in patient tumor samples, it also enabled a patient outcome association that was not evident with regular selection criteria as shown by our data in Verhagen et al. [21]. Together our data also suggest that variant selection should not solely be guided by pathogenicity criteria (derived from cancer incidence data as by COSMIC annotation) since this results in a lack of hits in our repair defected HNSCC cell line panel. The potential impact on protein function of the selected variants will have to be evaluated in future studies. This is a massive endeavor considering the multitude of variants and questionable in value due to the infrequency of the individual variants. This points to the importance of variant selection strategies that provide an increased link to functional endpoints on a cellular level so to be able to perform valuable outcome association studies.

## Conclusion

Assuming to be a prerequisite but without proof of concept, homozygocity of DNA repair gene variants has been used to mark tumor samples with potential defects. Here we tested this in the genetic context of tumor cells and show its benefit and association with repair endpoints. The inclusion of rare homozygous SNPs to mark potentially repair defected cell lines further improves this association and highlights their potential role in genomic studies. We conclude that allele zygosity and SNP allele frequency selection criteria can be used to identify FA/HR repair-defected samples and relevant genes.

## Supporting information

S1 File **Figure A**. Illustration of MMC response and pathway mutation association testing. **Figure B**. Barplot of MMC IC_50_ values in the cell line panel. **Figure C**. Density plot of VAF values of variants in cell line panel and control blood samples. **Figure D**. Effect of VAF thresholds on the number of FA/HR variants, number of FA/HR mutated cell lines, and difference in MMC IC_50_ values of FA/HR mutated and non-mutated cell lines. **Figure E**. Wilcoxon rank-sum test P-values comparing the MMC IC_50_ values of FA/HR mutated and non-mutated cell lines as a function of VAF thresholds. **Figure F**. Scatterplot number of variants against MMC sensitivity. **Figure G**. Scatterplot number of homozygous variants against MMC sensitivity. **Figure H**. Effect of MAF thresholds on the number of FA/HR variants, number of FA/HR mutated cell lines, and difference in MMC IC_50_ values of FA/HR mutated and non-mutated cell lines. **Figure I**. Wilcoxon rank-sum test P-values comparing the MMC IC_50_ values of FA/HR mutated and non-mutated cell lines as a function of MAF thresholds. **Figure J**. Selection of high VAF variants results in a significant functional endpoint association with multiple MAF thresholds. **Figure K**. Selection of low MAF SNPs and non-SNP variants results in a significant functional endpoint association when focusing on those with a high VAF. **Figure L**. Volcano plot of associations between MMC response and KEGG pathways. **Figure M**. PPV and Wilcoxon rank-sum test P-values for MMC sensitivity association analyses with variants selected based on REVEL and CADD deleteriousness scores. **Figure N**. Volcano plot of associations between MMC response and KEGG pathways based on REVEL and CADD based variant selection. **Figure O**. Density plot of VAF values of variants in HNSCC tumor samples and control blood samples. **Table A**. Capture set and genes comprising the canonical FA/HR gene set. **Table B**. Cell line *TP53* mutation status according to literature and our variant calling. **Table C**. Established and candidate cancer genes in HNSCC.
(PDF)
