# Supplementary material for: Role of variant allele fraction and rare SNP filtering to improve cellular DNA repair endpoint association
Source: PLoS One. 2018 Nov 8;13(11):e0206632. doi: 10.1371/journal.pone.0206632 (PMC6224072; doi:10.1371/journal.pone.0206632)
Supplement: S1 File — Figure A. Illustration of MMC response and pathway mutation association testing. Figure B. Barplot of MMC IC50 values in the cell line panel. Figure C. Density plot of VAF values of variants in cell line panel and control blood samples. Figure D. Effect of VAF thresholds on the number of FA/HR variants, number of FA/HR mutated cell lines, and difference in MMC IC50 values of FA/HR mutated and non-mutated cell lines. Figure E. Wilcoxon rank-sum test P-values comparing the MMC IC50 values of FA/HR mutated and non-mutated cell lines as a function of VAF thresholds. Figure F. Scatterplot number of variants against MMC sensitivity. Figure G. Scatterplot number of homozygous variants against MMC sensitivity. Figure H. Effect of MAF thresholds on the number of FA/HR variants, number of FA/HR mutated cell lines, and difference in MMC IC50 values of FA/HR mutated and non-mutated cell lines. Figure I. Wilcoxon rank-sum test P-values comparing the MMC IC50 values of FA/HR mutated and non-mutated cell lines as a function of MAF thresholds. Figure J. Selection of high VAF variants results in a significant functional endpoint association with multiple MAF thresholds. Figure K. Selection of low MAF SNPs and non-SNP variants results in a significant functional endpoint association when focusing on those with a high VAF. Figure L. Volcano plot of associations between MMC response and KEGG pathways. Figure M. PPV and Wilcoxon rank-sum test P-values for MMC sensitivity association analyses with variants selected based on REVEL and CADD deleteriousness scores. Figure N. Volcano plot of associations between MMC response and KEGG pathways based on REVEL and CADD based variant selection. Figure O. Density plot of VAF values of variants in HNSCC tumor samples and control blood samples. Table A. Capture set and genes comprising the canonical FA/HR gene set. Table B. Cell line TP53 mutation status according to literature and our variant calling. Table C. Established and candidate cancer gene [file pone.0206632.s001.pdf]

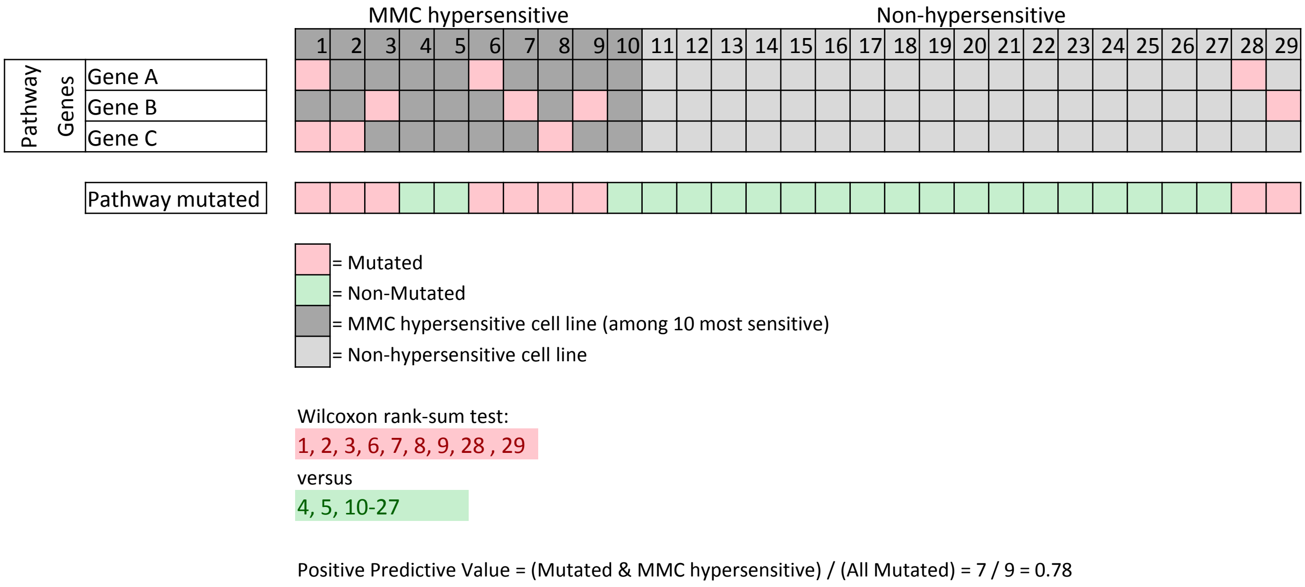

Figure A

Illustration of the association analysis between pathway variants and MMC response. We compared ‘pathway-mutated’ with ‘non-mutated’ cell lines. ‘Pathway-mutated’ cell lines had one or more variants in any of the pathway genes under investigation (pink), the ‘non-mutated’ had none (green). The Wilcoxon rank-sum test was used to compare the (ranked) MMC IC_{50} values of ‘pathway mutated’ and ‘non-mutated’ cell lines. The PPV was calculated as the ratio of true positive calls over all positive calls. Positive calls are ‘pathway-mutated’ cell lines as marked by the different variant selection criteria; true positive calls were ‘pathway-mutated’ cell lines that were also among the ten most MMC sensitive cell lines (hypersensitive cell lines in dark grey).

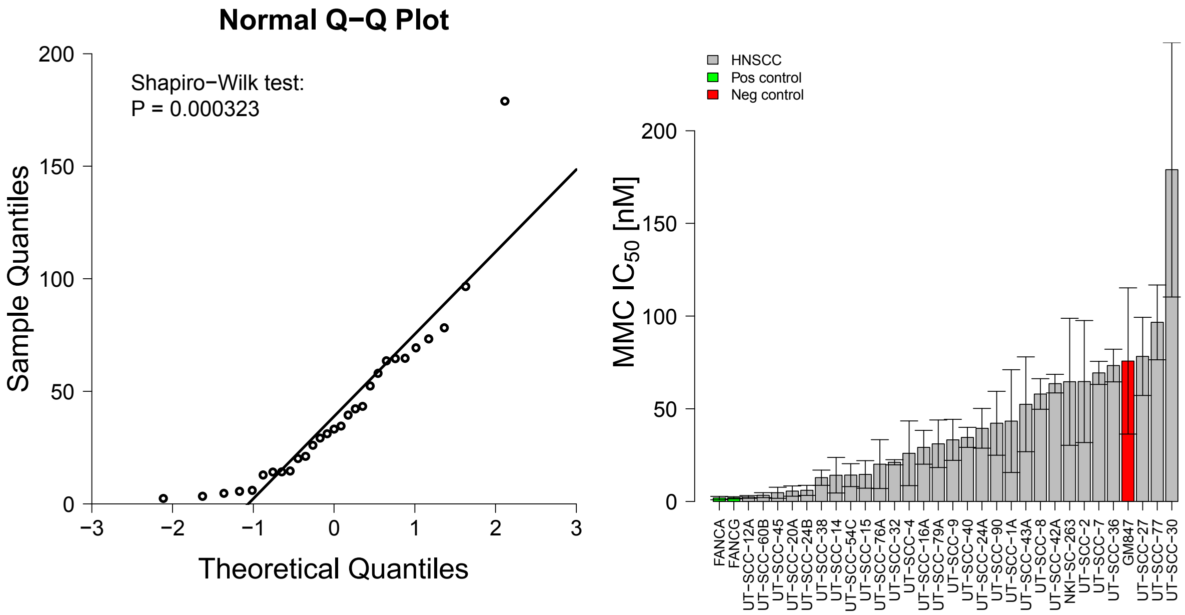

Figure B

MMC IC_{50} values distribution in the HNSCC cell line panel. (a) The distribution of the MMC IC_{50} values (as previously reported by us in Verhagen et al. [23] was assessed. The inlet Q-Q plot and stated significant Shapiro-Wilk test illustrates the non-normal distribution of the HNSCC cell line MMC IC_{50} values. (b) MMC IC_{50} values. The positive control cell lines with confirmed FA gene defects are depicted in green (FANCA, FANCG), the negative control normal fibroblast line in red. MMC response of the HNSCC cell lines (grey bars) varied from hypersensitive (similar sensitivity as the positive control) to normal sensitivity (similar sensitivity as the negative control). Values are calculated from the curve-fits on the individual experiment data and are the average of three to five independent experiments; bars are SEM.

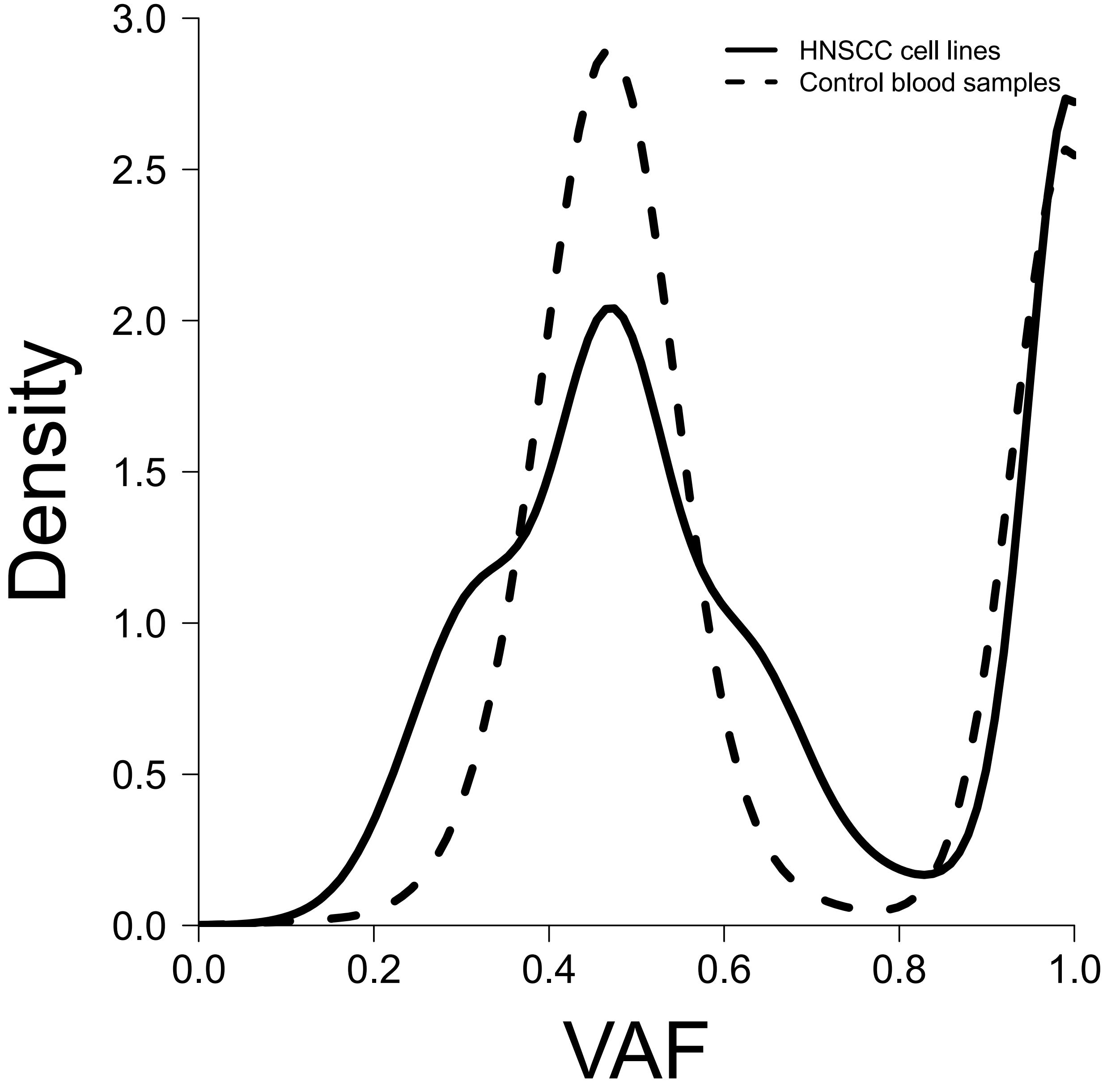

Figure C

Average allele zygosity is reflected in the VAF distribution of variants. Density plot of the VAF distribution of all non-synonymous exonic and splice site variants in all captured genes (both SNP and non-SNP). The VAF distributions of the HNSCC cell line panel and control blood samples both show a bimodal distribution of hetero- and homozygous variants; the latter shows a narrower distribution. The minimum in the curve appears around 0.8. The threshold for homozygous variants was therefore set at 0.8.

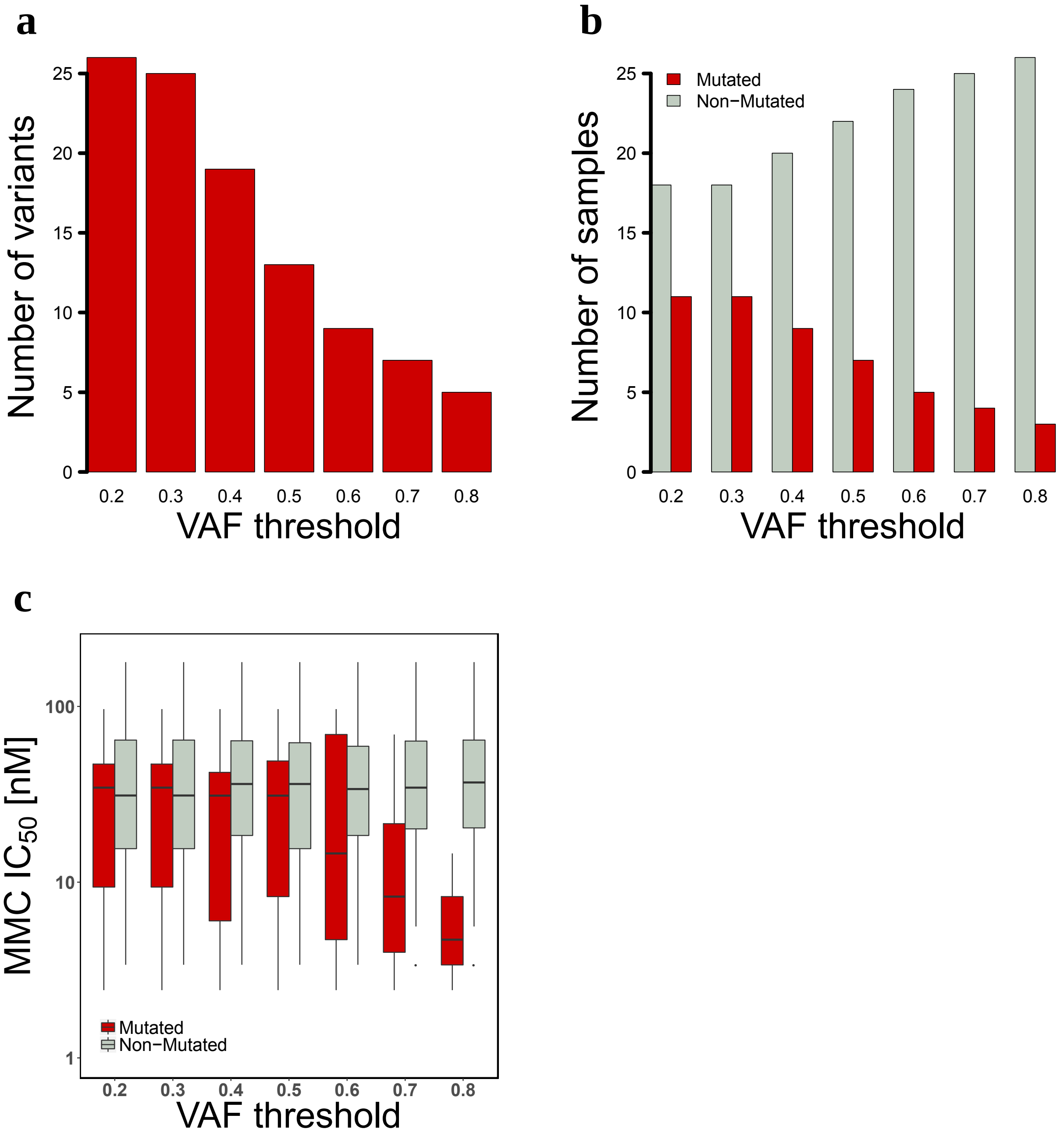

Figure D

The effect of VAF filtering on the number of FA/HR gene variants and 'mutated' cell line calling. We varied VAF thresholds from low to high and at each threshold value removed variants with a VAF below the threshold. At each VAF threshold we depict (a) the total number of variants exceeding the threshold, (b) the number of cell lines with a FA/HR variant exceeding the threshold ('mutated'), and (c) the MMC IC_{50}s of the FA/HR 'mutated' and 'non-mutated' cell lines at the indicated thresholds.

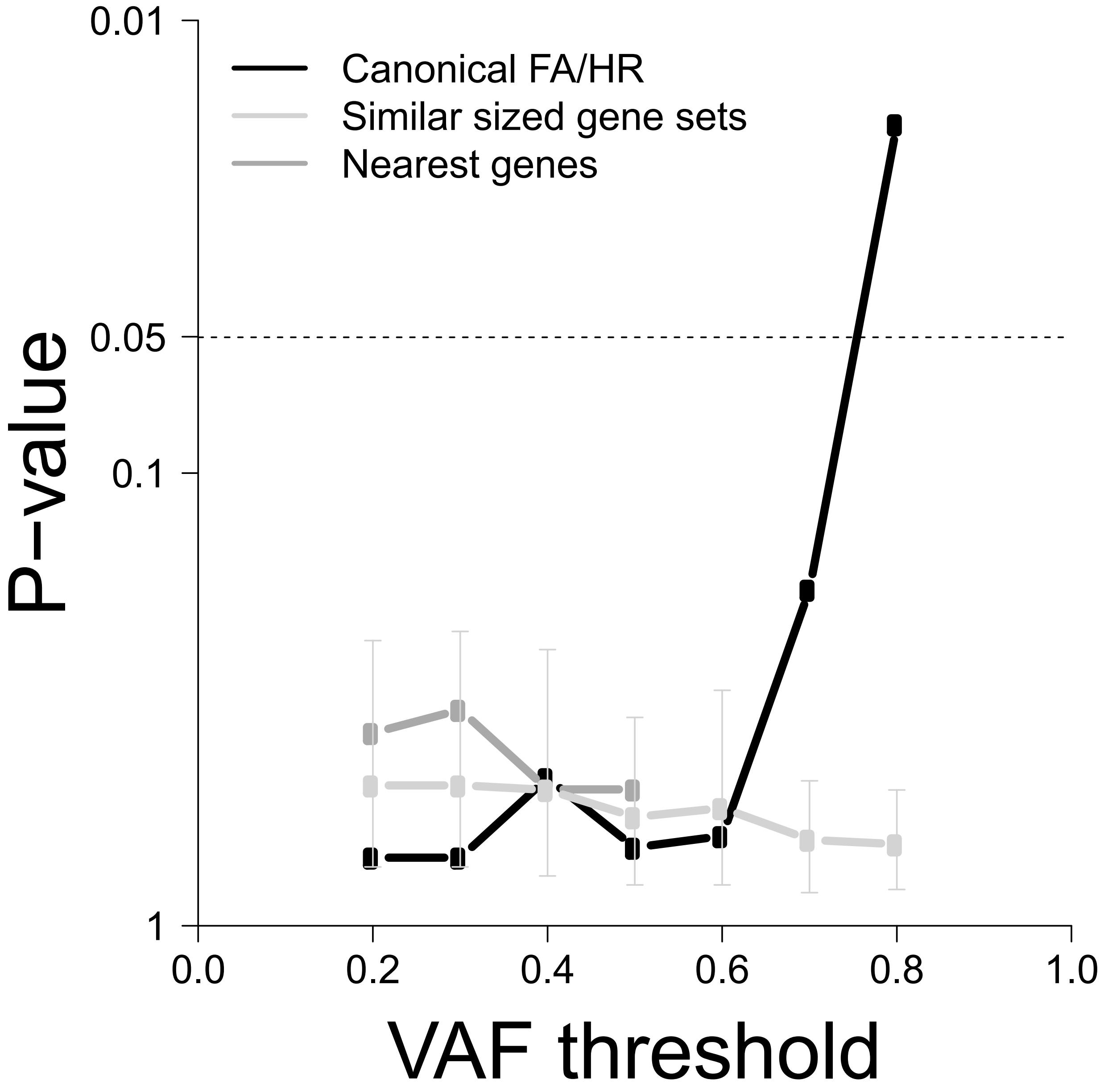

Figure E

Selection of high VAF variants improves functional, MMC response, association of selected cell lines. Association of MMC sensitivity with increasing VAF thresholds for variant selection. Wilcoxon rank-sum test *p*-values are shown*. MMC response associations are shown for cell lines marked by canonical FA/HR gene set variants (excluding SNPs black solid line, including SNPs black dashed line) and control gene sets. The control gene sets are 10.000 randomly grouped similar-sized gene sets (light grey solid line). The with error bars extend from the median to the first and third quartiles. Dark grey solid line depicts the results for the nearest gene set.

* Statistical tests were not performed at thresholds that resulted in less than two or all samples to be marked by a variant. Thin dashed horizontal line shows significance level of 0.05.

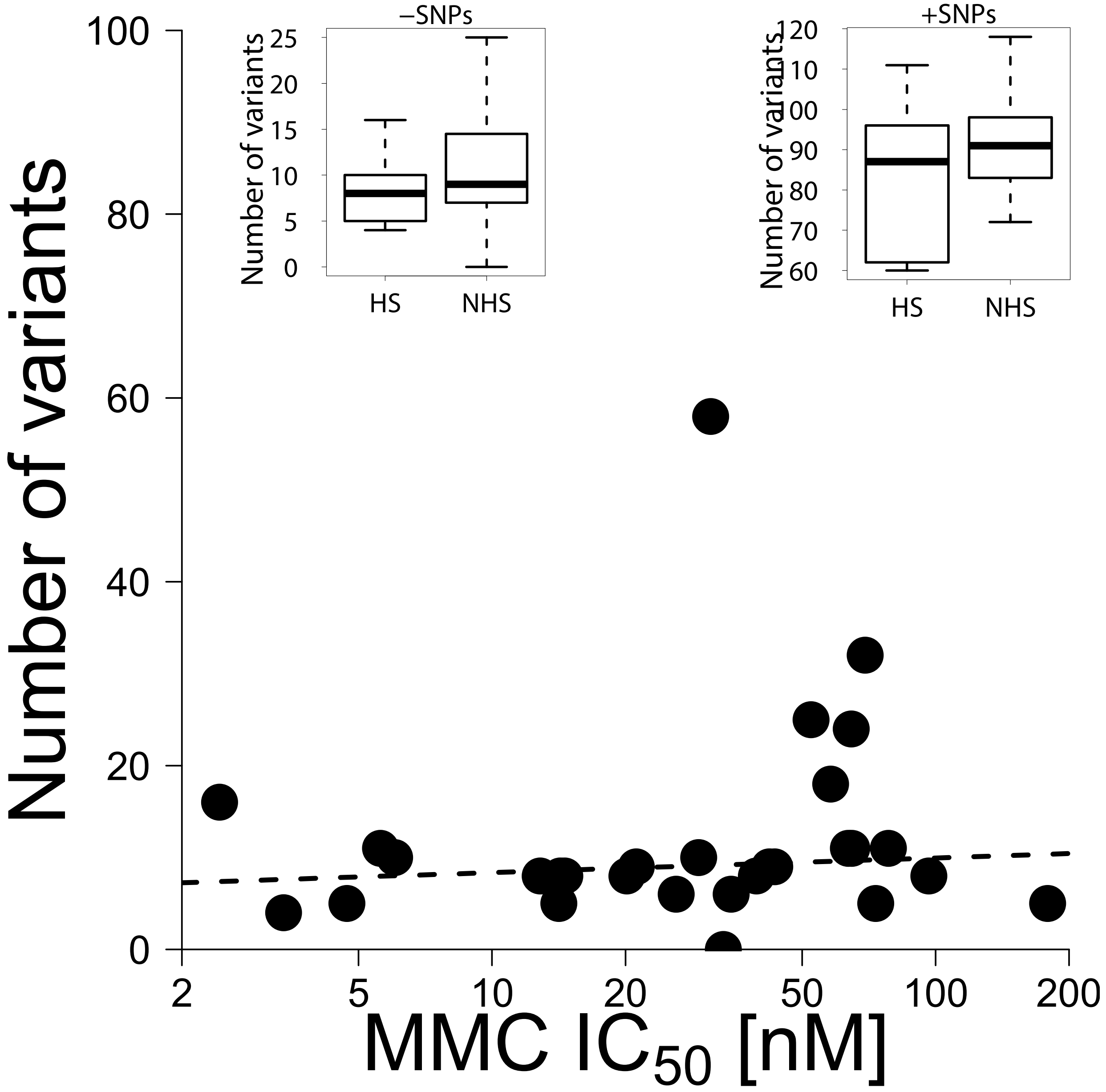

Figure F

Variant load in the cell lines is not associated with MMC sensitivity. The total number of variants in all captured and sequenced genes, as obtained by applying the bioinformatics pipeline described in Materials and Methods and after removing all SNPs, were calculated for each cell line and plotted against the MMC IC_{50} value of the respective cell line. Dashed black line shows the regression model with MMC sensitivity as a covariate ($p = 0.81$). The inlets show the average variant load as determined above in the hypersensitive cell lines (HS) and non-hypersensitive lines (NHS) both excluding (-) or including (+) SNPs.

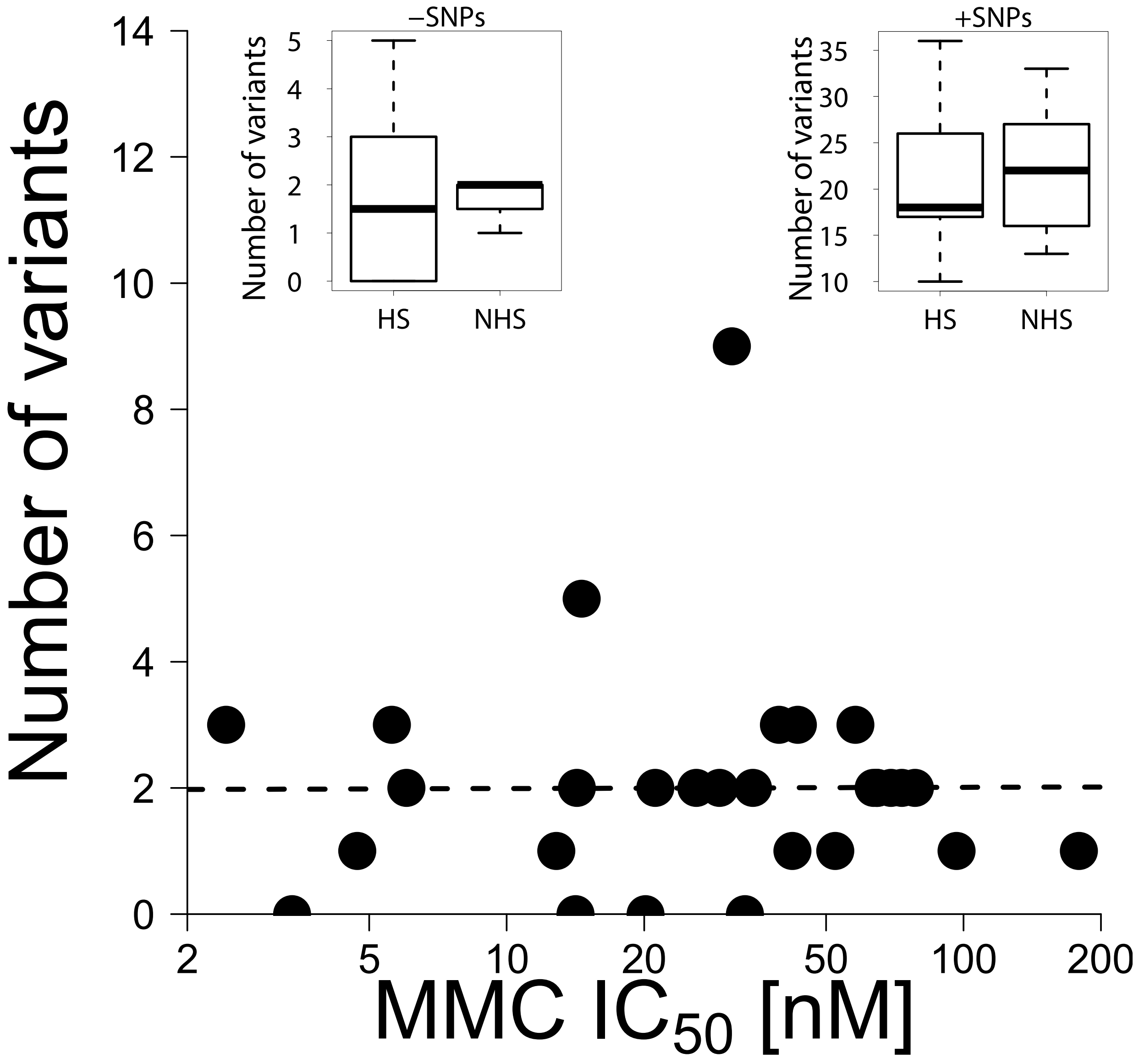

Figure G

Homozygous variant load in the cell lines is not associated with MMC sensitivity. The number of homozygous variants in all captured and sequenced genes as obtained by applying the bioinformatics pipeline described in Materials and Methods and after removing all SNPs. Dashed black line shows the regression model with MMC sensitivity as a covariate (p = 0.65). The inlets show the average homozygous variant load as determined above in the hypersensitive cell lines (HS) and non-hypersensitive lines (NHS) either excluding (-) or including (+) SNPs.

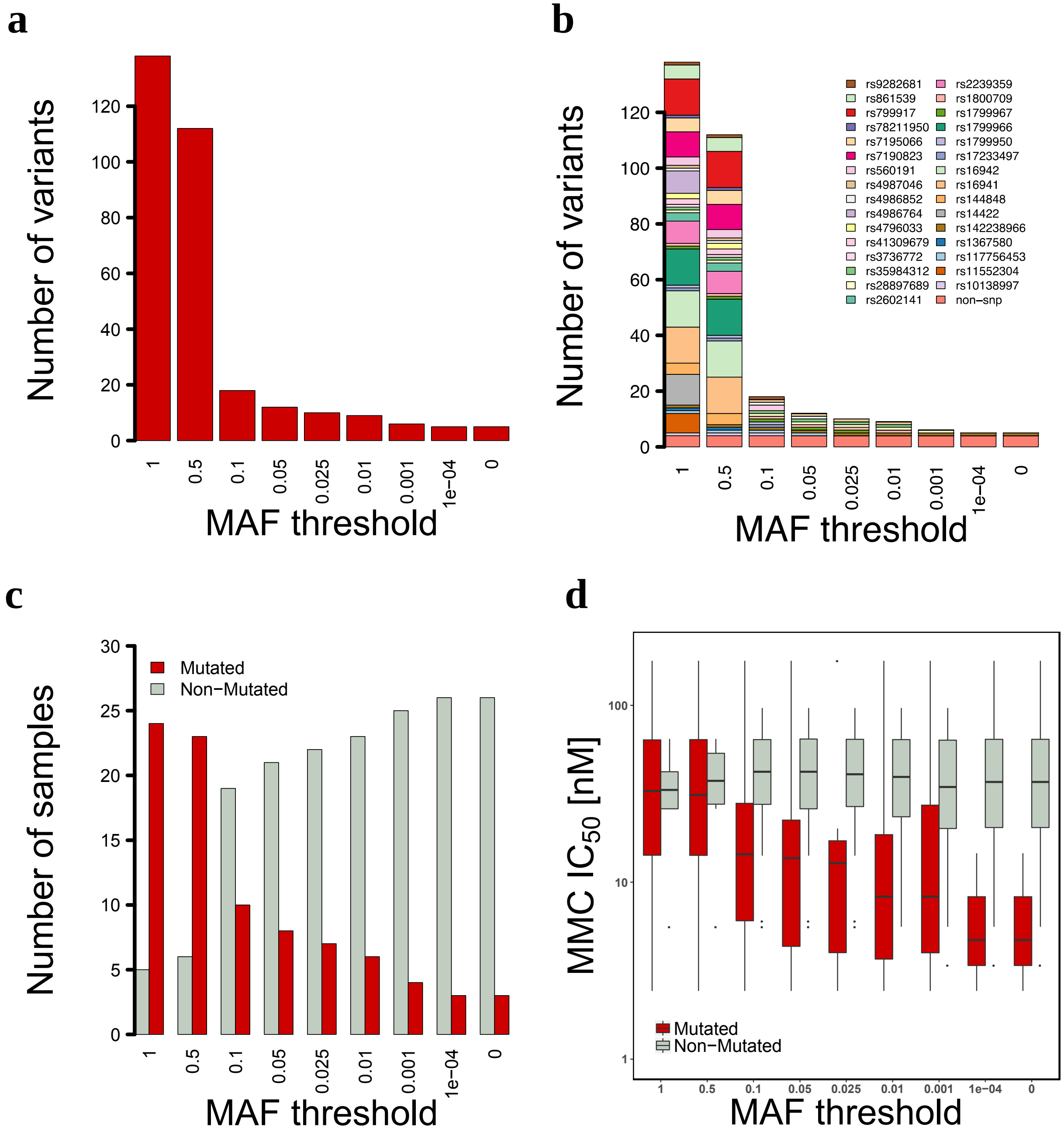

Figure H

The effect of MAF filtering on the number of selected FA/HR gene variants and mutated cell lines. Focusing on all homozygous FA/HR variants (VAF ≥ 0.8), MAF thresholds were varied and at each threshold value SNPs with a MAF below the threshold were removed. The total number of variants and SNPs with MAF below the threshold (a), the split according to dbSNP identifier (b), the number of cell lines with a FA/HR gene variant or SNP with MAF below the threshold ('mutated') (c), and (d) the MMC IC_{50}s of the cell lines assigned to be FA/HR 'mutated' and 'non-mutated' according to the presence of a variant selected by these criteria are shown at each MAF threshold. Stars indicate significance levels.

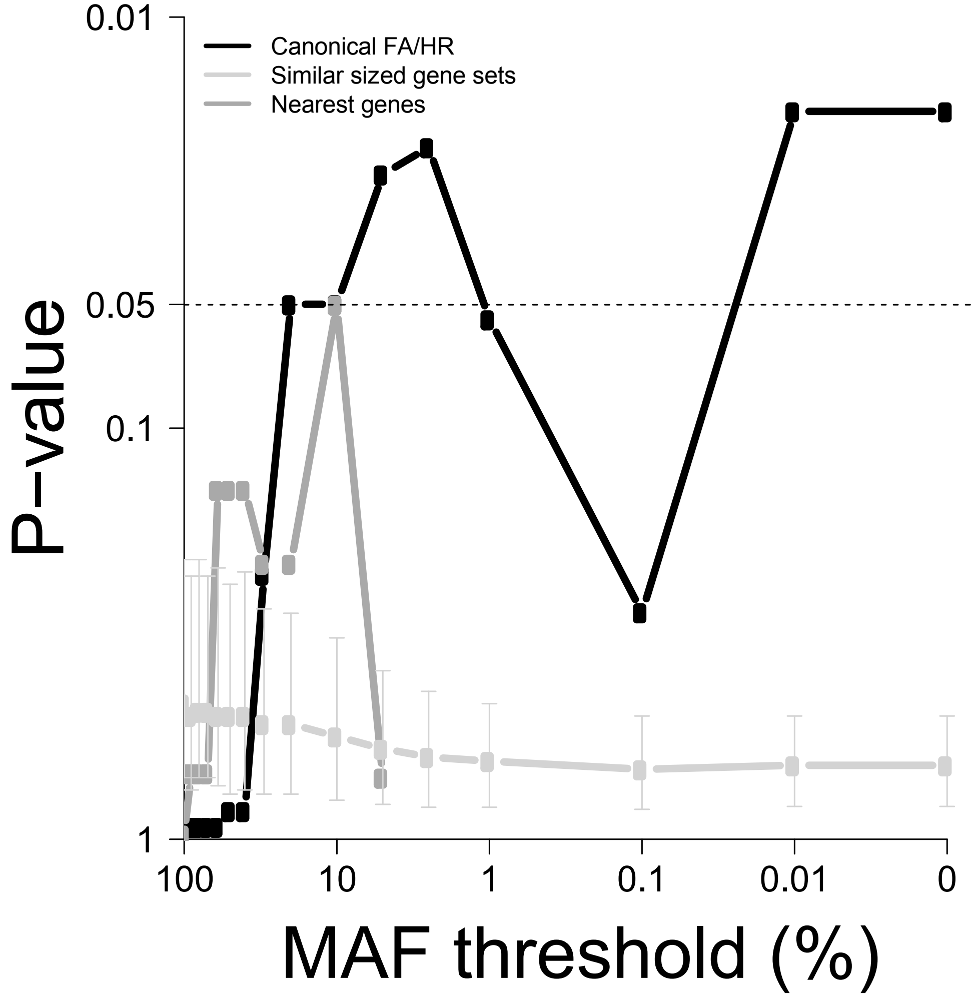

Figure I

Selecting low MAF SNPs and non-SNP variants improves functional, MMC response, association of selected cell lines. Association of variants with MMC sensitivity as determined by the Wilcoxon rank-sum test *p*-value* in the canonical FA/HR gene set (black solid line) and control gene sets (light grey solid line for the 10.000 similar sized gene sets with errors bars extending from the median to the first and third quartiles, dark grey solid line for the nearest genes) with decreasing MAF thresholds.

* Statistical tests were not performed at thresholds that resulted in less than two or all samples to be marked by a variant. Thin dashed horizontal line shows significance level of 0.05.

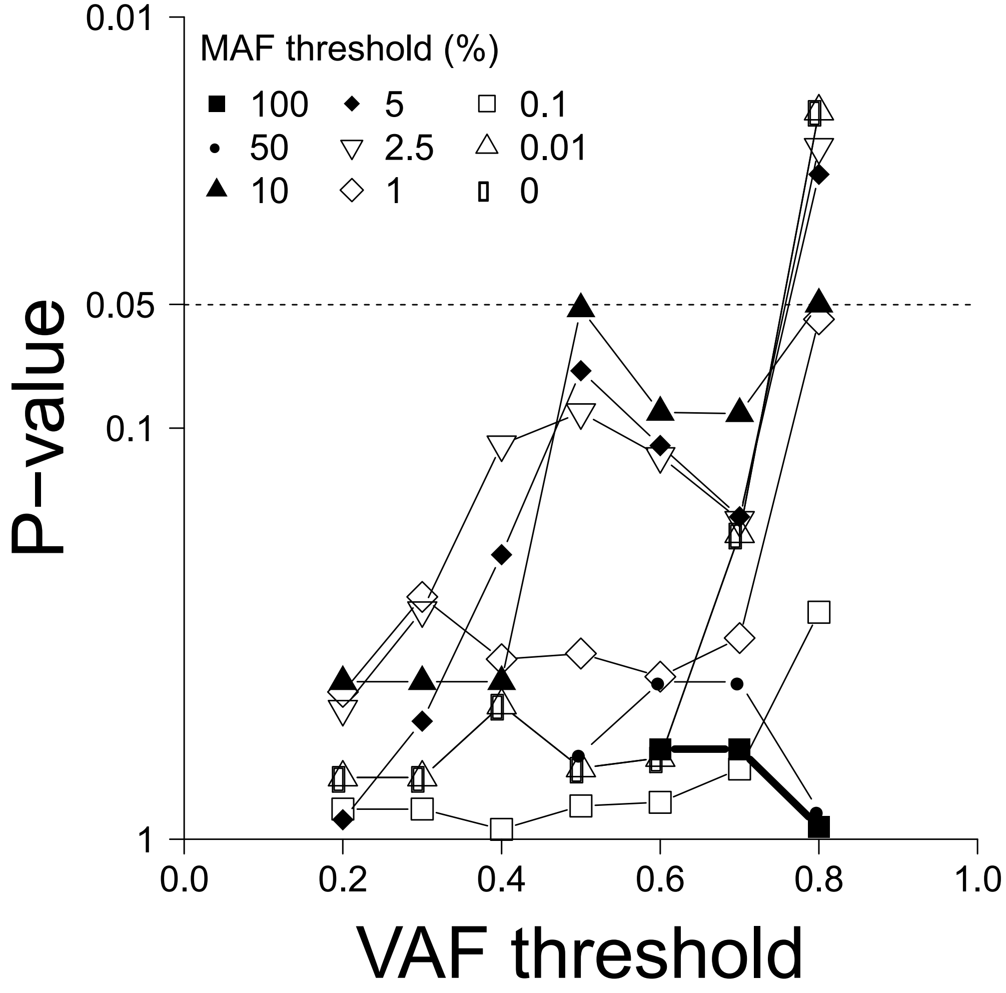

Figure J

Selection of high VAF variants results in a significant functional endpoint association with multiple MAF thresholds. Association of canonical FA/HR gene set variants with MMC sensitivity. Wilcoxon rank-sum test *p*-values* with increasing VAF thresholds are shown. Figure shows this analysis for different MAF thresholds above which SNPs were excluded.

* Statistical tests were not performed at thresholds that resulted in less than two or all samples to be marked by a variant. Thin dashed horizontal line shows significance level of 0.05.

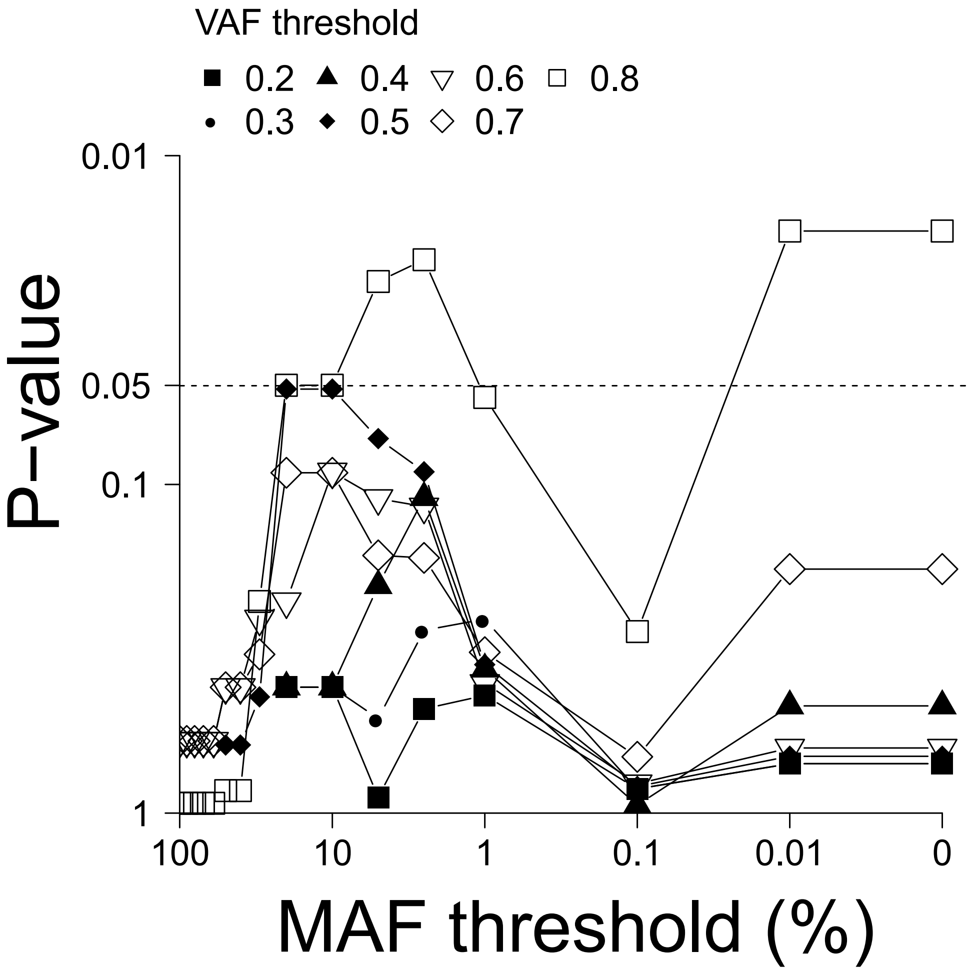

Figure K

Selection of low MAF SNPs and non-SNP variants results in a significant functional endpoint association when focusing on those with a high VAF. Association of canonical FA/HR gene set variants with MMC sensitivity. Wilcoxon rank-sum test *p*-values* with decreasing MAF thresholds are shown. Different VAF thresholds, excluding variants with a VAF below these thresholds, as indicated above were tested. Significant associations are found at multiple MAF thresholds, but only when the analysis is restricted to homozygous variants (VAF ≥ 0.8).

* Statistical tests were not performed at thresholds that resulted in less than two or all samples to be marked by a variant. Thin dashed horizontal line shows significance level of 0.05.

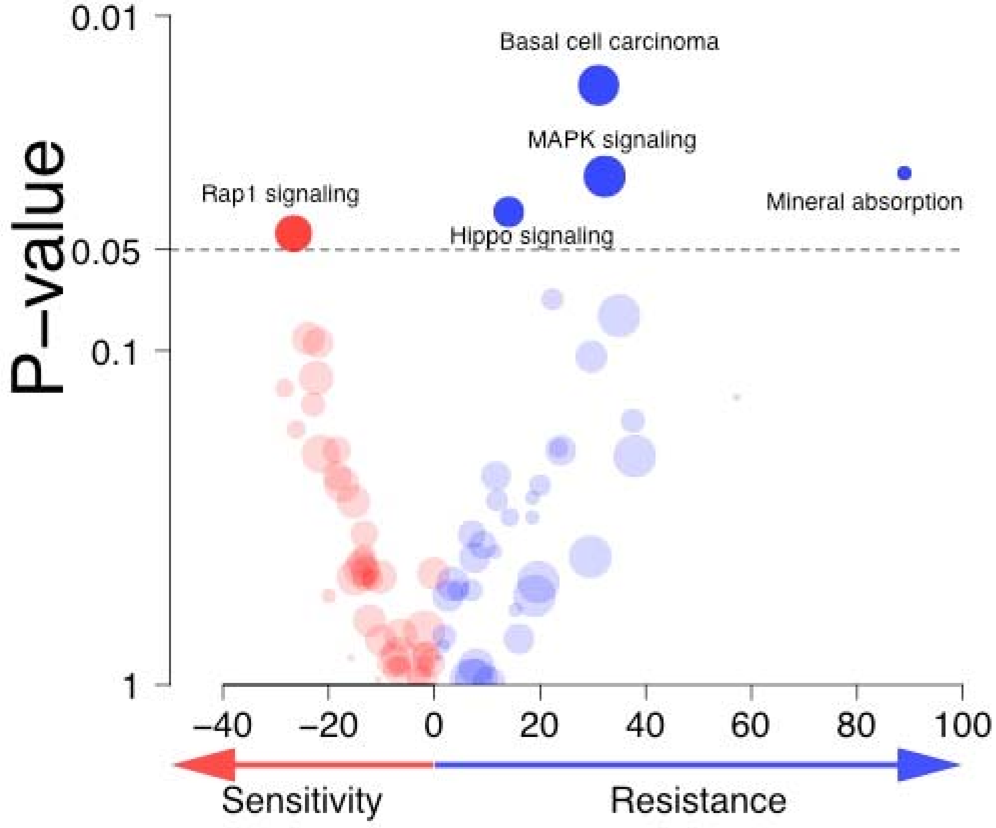

Figure L

Volcano plot MMC response and individual KEGG pathways associations (without *TP53* mutation status correction). The x-axis is the difference in mean MMC IC_{50} between the cell lines that were called ‘pathway mutated’ and ‘non-mutated’. The y-axis shows the significance of this difference (Wilcoxon rank-sum test). ‘Pathway-mutated’ cell lines are those with one or more variants in any gene of the individual KEGG pathway. Dot size is proportional to the number of ‘pathway mutated’ cell lines.

Note: the association of MMC with the MAPK signaling and basal cell carcinoma pathways was erroneous due to missed *TP53* mutations in 2 out of 10 MMC hypersensitive cell lines (see main text and Table C).

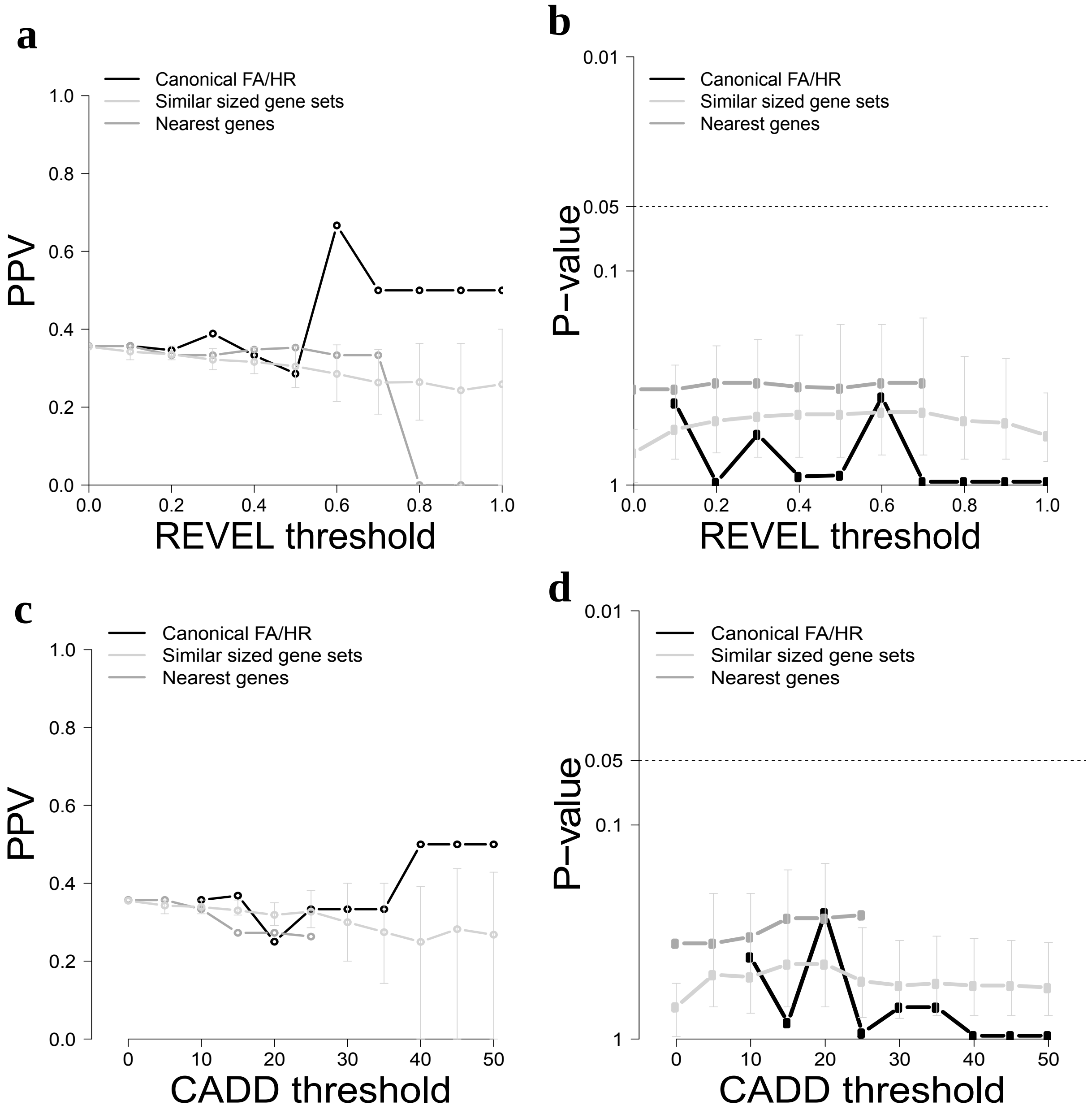

Figure M

Variant selection based on REVEL and CADD deleteriousness scores and association with MMC sensitivity. We varied REVEL and CADD score thresholds from low to high and at each threshold value removed variants with a score below the threshold. Variants with REVEL scores > 0.5 and CADD scores > 15 are generally considered deleterious. Although in-frame and frameshift indels and nonsense mutations aren't scored by REVEL or CADD, these were considered as potentially pathogenic and therefore included at each threshold.

The positive predictive value (PPV) for MMC sensitivity was used to quantify the ability of variants to label repair defected cell lines (i.e. ten most MMC sensitive) *. **(a)** and **(c)** PPV values at each REVEL and CADD threshold. **(b)** and **(d)** association of variants with MMC response as determined by the Wilcoxon rank-sum test P-value.

* Statistical analyses were omitted at sample sizes of a group of less than two and these data points and lines have been excluded in the figures.

a

b

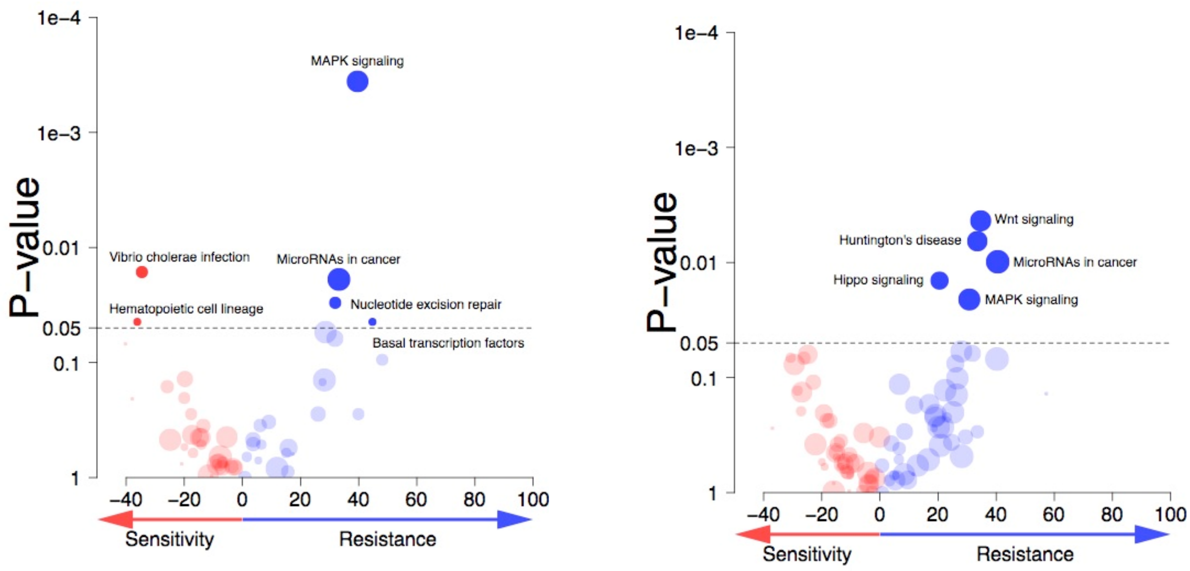

Figure N

Volcano plot MMC response and individual KEGG pathways associations after REVEL (A) and CADD (B) variant selection and based on 'deleteriousness' prediction. The x-axis is the difference in mean MMC IC_{50} between 'pathway mutated' and 'non-mutated' cell lines. The y-axis shows the significance of the difference in MMC IC_{50} between pathway mutated and non-mutated cell lines (Wilcoxon rank-sum test). Pathway mutated cell lines are those with one or more variants in any gene of the individual KEGG pathway. Dot size is proportional to the number of 'pathway mutated' cell lines. Variants with **(a)** a REVEL score > 0.5 and **(b)** a CADD score > 15 were retained, as these are generally considered pathogenic. Although in-frame and frameshift indels and nonsense mutations aren't scored by REVEL or CADD, we considered these pathogenic and included them at each threshold.

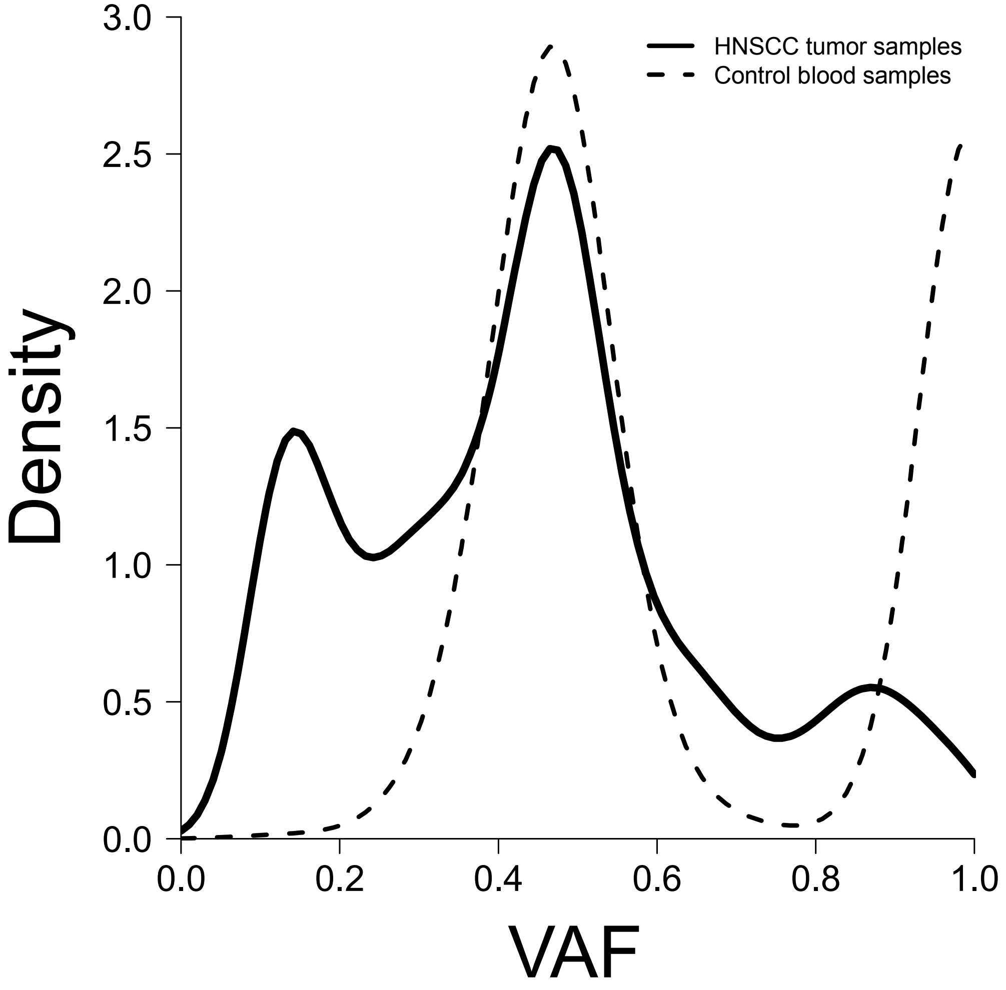

Figure O

VAF density plot of variants in 56 HNSCC tumor samples. Variants were called with the same variant calling pipeline that was used for the HNSCC cell line panel. Only rare SNPs were included (MAF ≤ 1%). The VAF density of variants in the HNSCC tumor samples has three peaks. In contrast, the density function of variants in control blood samples has a bimodal distribution reflecting the hetero- and homozygous states.

I

Capture composition
Genes involved in DNA damage response
Homologous Recombination
Fanconi Anemia pathway
Non Homologous End Joining
Nucleotide Excision Repair
Base Excision Repair
Mismatch Repair
Translesion Synthesis
Genes involved in cell cycle regulation and proliferation
Genes involved in drug activation and transport
Genes involved in drug sensitivity
cisplatin
MMC
PARP inhibitors
Frequently mutated genes in cancer, in particular:
Head and Neck Cancers (HNSCC)
Lung Cancer (NSCLC)
Breast Cancer
Colon Cancer

II

Canonical FA/HR gene set	Matched nearest genes
FANCA	*CDT1*
FANCB	*POLA1*
FANCC	*PTCH1*
BRCA2	*RFC3*
FANCD2	*VHL*
FANCE	*CDKN1A*
FANCF	*ABCC8*
FANCG	*APTX*
FANCI	*POLG*
BRIP1	*PPM1D*
FANCL	*PSME4*
FANCM	*POLE2*
PALB2	*PLK1*
RAD51C	*AKAP1*
SLX4	*ABCA3*
RAD51	*LTK*
TP53BP1	*LTK*
BRCA1	*TOP2A*
C17orf70	*CSNK1D*
RAD50	*PPP2CA*
RAD51B	*HIF1A*
RAD51D	*LIG3*
RAD52	*FOXM1*
RAD54B	*NBN*
RAD54L	*MUTYH*
XRCC3	*AKT1*
XRCC2	*SMARCD3*

Table A

Composition of 556 gene capture set (I) and list of genes comprising the canonical FA/HR gene set (II) used in this study. **(I)** The primary objective in the selection of the capture genes was to cover DNA repair genes and genes reported to be relevant in head and neck squamous cell carcinoma (HNSCC). Cell cycle regulation and drug activation and transporter genes were selected since they influence crosslinker response, even though to a lesser degree. Genes mediating sensitivity to drugs such as cisplatin and PARPi, unrelated to DNA repair, were added. Moreover, we included frequently mutated genes in HNSCC (as of 2014) to be able to assess those in the HNSCC cell line panel. We broadened this selection with additional frequently mutated genes, as reported in other human cancers. **(II)** The matched nearest genes were selected from the 556 gene capture set to be closest to each canonical FA/HR gene, as measured by base pair distance.

Cell line	*TP53* literature	*TP53* DNA-seq	Reference	Comment
UT-SCC-12A	Mut	Wildtype	[33]	Read coverage of 4, therefore discarded by pipeline due to low coverage
UT-SCC-60b	Mut	Wildtype	[34]	Deletion of 25 base pairs
UT-SCC-45	Wildtype	Wildtype	[35]	Cell line from HPV positive tumor
UT-SCC-20A	Mut	Mut	[36]	
UT-SCC-24B	Mut	Mut	[33]	
UT-SCC-38	Unknown	Wildtype		
UT-SCC-14	Mut	Mut	[36]	Deletion of 30 base pairs documented, splice site variant detected
UT-SCC-54C	Mut	Mut	[34]	
UT-SCC-15	Mut	Mut	[33]	
UT-SCC-76A	Unknown	Mut		
UT-SCC-32	Mut	Mut	[33]	
UT-SCC-4	Mut	Mut	[33]	
UT-SCC-16A	Mut	Mut	[36]	
UT-SCC-79A	Unknown	Mut		
UT-SCC-9	Mut	Wildtype	[33]	Deletion of exons 2-9
UT-SCC-40	Mut	Wildtype	[22]	Deletion of 30 base pairs
UT-SCC-24A	Mut	Mut	[33]	
UT-SCC-90	Unknown	Wildtype		
UT-SCC-1A	Mut	Mut	[36]	
UT-SCC-43A	Mut	Mut	[34]	
UT-SCC-8	Mut	Mut	[22]	
UT-SCC-42A	Mut	Mut	[34]	
NKI-SCC-263	Unknown	Mut		
UT-SCC-2	Mut	Mut	[36]	
UT-SCC-7	Mut	Mut	[33]	
UT-SCC-36	Mut	Mut	[22]	
UT-SCC-27	Mut	Mut	[22]	
UT-SCC-77	Mut	Mut	[37]	
UT-SCC-30	Mut	Mut	[36]	

Table B

List of cell lines and their *TP53* mutation status in the literature and as called by our DNA-seq pipeline. Literature references and a comment explaining the discrepancy are given between the literature. Five reported *TP53* mutations were missed. The mutation in UT-SCC-12A was present in the raw reads, but not called by VarScan due to low sequencing coverage. The other four comprised medium-sized deletions and were missed due to the short-read DNA-sequencing approach that is not well suited to detect deletions of this size. Importantly, two of these missed mutations were among the most MMC sensitive cell lines. This resulted in false associations between *TP53* mutation status and MMC response and a false association with the MAPK signaling and basal cell carcinoma pathways in the pathway analyses (Figure L). These associations disappeared when correcting *TP53* mutation status to the reported (Fig 2B).

Gene	Function	Capture sequenced
FHIT	Tumor suppressor	No
RASSF1A	Tumor suppressor	No
CSMD1	Tumor suppressor	Yes
CDKN2A	Tumor suppressor	Yes
PTPRD	Tumor suppressor	Yes
PTEN	Tumor suppressor	Yes
TP53	Tumor suppressor	Yes
SMAD4	Tumor suppressor	Yes
CCNL1	Oncogene	Yes
PARP1	Oncogene	Yes
PIK3CA	Oncogene	Yes
TP63	Oncogene	Yes
DCUN1D1	Oncogene	Yes
EGFR	Oncogene	Yes
MET	Oncogene	Yes
MYC	Oncogene	Yes
PTK2	Oncogene	No
CCND1	Oncogene	Yes
CTTN	Oncogene	Yes
FADD	Oncogene	No

Table C

List of established and candidate cancer genes in HNSCC as described and taken from Leemans et al. 2011 [40]. The analysis was narrowed to the genes that were captured sequenced (third column: ‘Yes’).
